# Commentary: CRISPR–Cas Encoding of a Digital Movie into the Genomes of a Population of Living Bacteria

**DOI:** 10.3389/fbioe.2017.00057

**Published:** 2017-09-27

**Authors:** Ianis G. Matsoukas

**Affiliations:** ^1^School of Sport and Biomedical Sciences, The University of Bolton, Bolton, United Kingdom

**Keywords:** biotechnology, chemical biology, clustered regularly interspaced short palindromic repeats, DNA, genome engineering, synthetic biology, synthetic DNA

## Introduction

Clustered regularly interspaced short palindromic repeats (CRISPR) is a term that has become synonymous with genome editing. CRISPR enables researchers to modify genomic DNA *in vivo* directly and efficiently. Several review articles have been published on the history, biotechnology, and implications of CRISPR system recently (Doudna and Charpentier, 2014; Zhang et al., 2014; Barrangou, 2015; Lander, 2016; Ledford, 2016), so the CRISPR biotechnology will not be described in great detail here.

The foundational discoveries that led to CRISPR biotechnology can be traced back to 1993 (Mojica et al., 1993), when the genomic regions known as CRISPR loci were first identified. In 2007, after years of studying CRISPR genetic motifs, Barrangou et al. (2007) came to the conclusion that CRISPR’s function is related to microbial cellular immunity. CRISPR identifies, targets, and eliminates foreign DNA. When a bacteriophage infects a bacterium, CRISPR cuts out fragment of the foreign DNA and stores it in the bacteria’s own genome. The bacterium then uses the stored DNA to recognize the virus and defend against future attacks. Since the discovery of the mechanism of action utilized by the CRISPR-associated (Cas) locus system, several different forms of the Cas loci have been characterized. While CRISPR–Cas system is revolutionary due to its speed and adaptability, it is not the first technology to enable genome engineering. That distinction belongs to a biotechnology known as zinc-finger nucleases (Bibikova et al., 2001). Other core technologies that commonly used to facilitate genome editing are the transcription activator-like effector nucleases (Boch et al., 2009; Moscou and Bogdanove, 2009), and homing endonucleases or meganucleases (Silva et al., 2011; Stoddard, 2014). However, the ease of use and versatility of CRISPR–Cas system has led to its rapid and broad adoption for genome engineering.

## Encoding a Movie into the DNA of Living Bacteria

Shipman et al. (2017) have recently described an experimental approach toward creating cellular recording systems that are capable of encoding a series of events. By combining the principles of information storage in DNA with DNA-capture systems capable of functioning in living cells, they created a bacterial system that capture, store, and propagate information over time. In 2016, the same group of scientists (Shipman et al., 2016) constructed the first molecular recorder based on the CRISPR system. The molecular recorder allows cells to acquire fragments of chronologically provided, DNA-encoded data that generate a memory in a bacterium’s genome.

**Figure 1:**
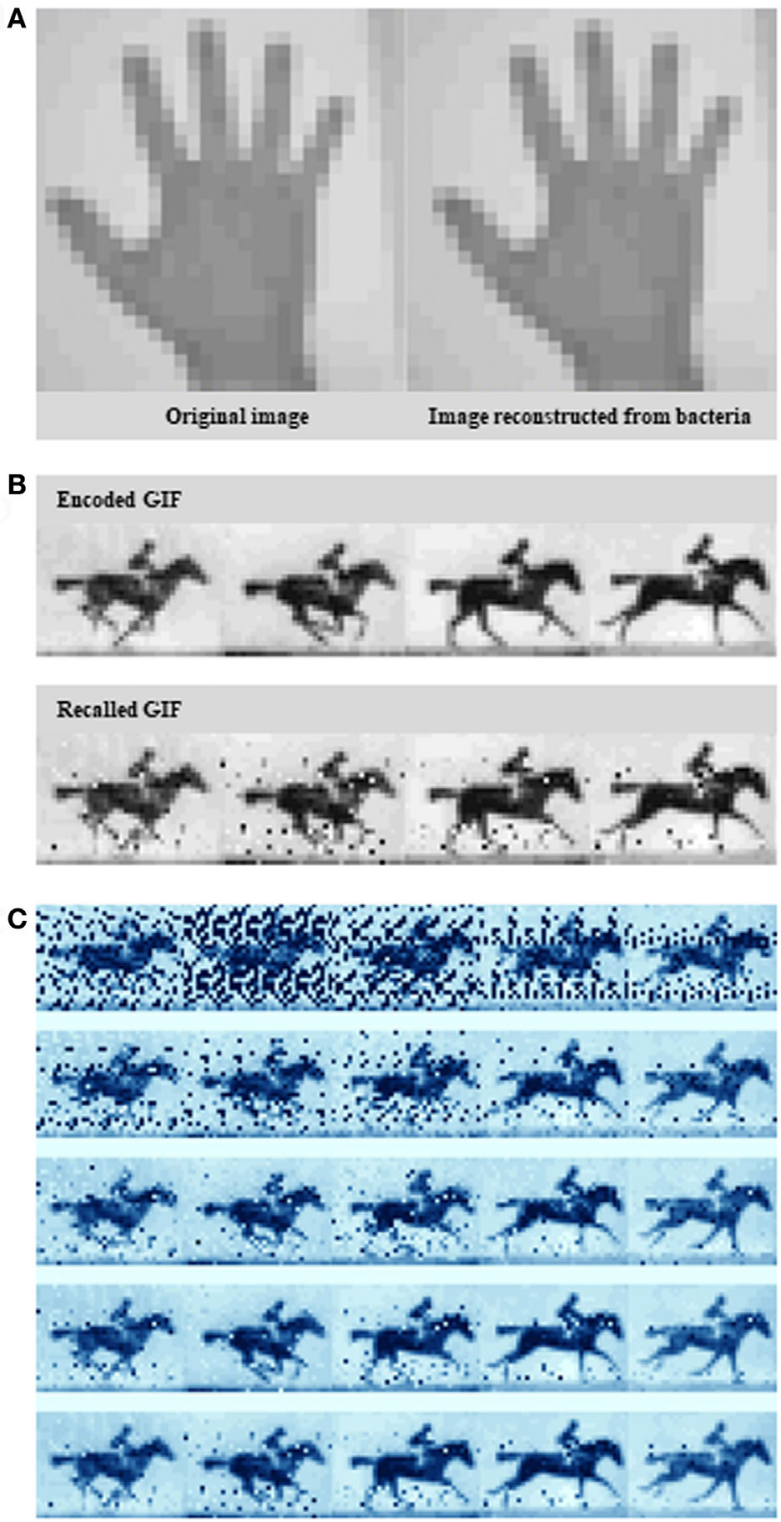
Encoding an image and a GIF into the genome. **(A)** A pixelated hand image. **(B)** A pixelated image of a galloping horse. **(C)** Exploiting the *Escherichia coli* type I–E clustered regularly interspaced short palindromic repeats–CRISPR-associated system to encode a primitive digital movie into—and then “play it back” from the bacterial genome. Examples of the output at different sequence depths. *Source*: Shipman et al. (2017).

In their recent article, Shipman et al. (2017) scale up this approach to define the information capacity that the system can record. Rather than arbitrary sequences, the novel bacterial system encoded real information such as a digitized image of a human hand (Figure 1A), reminiscent of some of the first paintings drawn on cave walls by early humans, and a sequence of five frames adapted from British photographer Eadweard Muybridge’s *Human and Animal Locomotion* series, that of a galloping horse (Figures 1B,C). The image represent constrained and clearly defined data sets, while the motion pictures, offer the opportunity to have bacteria acquire information frame-wise over time.

Clustered regularly interspaced short palindromic repeats genomic loci consist of repeat sequences, typically 20–50 bp in length, separated by variable spacer sequences of similar length (Bolotin et al., 2005; Mojica et al., 2005) that frequently match a fragment of foreign DNA. In prokaryotic viral defense mechanism, the Cas proteins, Cas1 and Cas2, function as an integrase complex to acquire nucleotides from invading viruses and store them in the CRISPR array (Barrangou et al., 2007; Nunez et al., 2014; Amitai and Sorek, 2016; Sternberg et al., 2016). During the process of integration, oligonucleotides of the foreign DNA, termed as a protospacer, is site-specifically incorporated into the host CRISPR locus as a new spacer at the leader-proximal end, where it serves as a molecular memory of prior infection (Barrangou et al., 2007; Deveau et al., 2008; Datsenko et al., 2012; Swarts et al., 2012; Yosef et al., 2012). However, the process of adaptation is not fully understood.

In the previous work, Shipman et al. (2016) provided evidence that the bacterial system could acquire synthetic sequences into the CRISPR array if those sequences are supplied as oligonucleotides. Interestingly, the integration of oligonucleotides into the CRISPR locus is non-random; the most recent viral elements are consistently integrated ahead of older viral elements in the array. Shipman et al. (2016) hypothesized that this temporal ordering of integration could form the basis of a molecular recording device. If defined synthetic DNA fragments could be integrated into CRISPR loci just as viral elements are, then sequencing the cells’ CRISPR loci would provide a record of which oligonucleotides the cells had been temporally and spatially exposed to. High-throughput sequencing has been an indispensable tool in targeted genome-editing biotechnologies. Interestingly, high-throughput sequencing has applications beyond simply sequencing genomes. Possibly one of the highest impact areas is the genome-wide deep mapping of regulatory elements at high resolution (Reuter et al., 2015; Goodwin et al., 2016).

In their recent article, Shipman et al. (2017) were able to uncover the underlying molecular principles of the CRISPR/Cas adaptation system, including sequence determinants of spacer acquisition that are relevant for understanding both the molecular mechanism of bacterial adaptation and its biotechnological applications. More specifically, their experimental strategy essentially translate the digital information contained in each pixel of an image or frame as well as the frame number into a DNA code, which, with additional sequences, is incorporated into spacers. This was achieved by exploiting the *Escherichia coli* type I–E CRISPR–Cas system.

## The Pixel Value-Coding and -Decoding Strategies

Shipman et al. (2017) encoded images of the human hand using two different pixel value-encoding strategies. First, they exploited the rigid encoding scheme, in which 4 pixel colors were each specified by a different base. They created several image protospacer sets by using a custom Python script to open and read the pixel values of the human hand image. Each protospacer was given a pixel code (a barcode that defined individual pixel sets) by a binary-to-nucleotide conversion, and populated by nucleotides encoding the pixel values according to the scheme detailed in the text. The pixel values encoded across the different protospacers then electroporated into a population of bacteria that overexpressed Cas1 and Cas2 to archive and propagate the human hand image data.

However, the rigid strategy did not work very well because it ended up generating some sequences that were not very compatible with the CRISPR system. In addition, Shipman et al. (2017) found that not all protospacer sequences were equally effective at transferring data into the genome. Hence, they ended up using a more flexible code, the flexible encoding scheme. The flexible strategy is similar to the codon code table used to build proteins. In this strategy, they had 21 colors and each color could be coded by three different nucleotide codes. Concisely, while the rigid encoding scheme is more dynamic since one pixel is defined by one base (whereas in flexible encoding scheme, one pixel is defined by one codon), the flexible encoding scheme is more suitable for obtaining more colored images, since there are more color options through increasing the number of bases in a codon. Finally, the original hand image was reconstructed by decoding the newly acquired spacers through high-throughput sequencing.

To create the galloping horse movie, Shipman et al. (2017) used a similar pixel value-encoding strategy. This time, they had to encode five images instead of one. More specifically, they translated five frames from the original racehorse movie into DNA, and over the course of 5 days they sequentially treated bacteria with frame after frame of translated DNA. Interestingly, it seems that Cas1 and Cas2 are the only Cas proteins required for new spacer acquisition at the host CRISPR locus (Datsenko et al., 2012; Yosef et al., 2012). Shipman et al. (2017) provided spacer collections for consecutive frames chronologically to a population of *E. coli* which, using Cas1/Cas2 activity, added them to the CRISPR arrays in their genomes. After retrieving all arrays, again from the bacterial population by high-throughput sequencing, they finally were able to reconstruct all frames of the galloping horse movie, and the order they appeared in with 90% accuracy (Figure 1C).

## Concluding Remarks

The interesting part of this research is not necessarily the image encoding but rather how Shipman et al. (2017) utilized the CRISPR system to integrate the encoding DNA into the genome of *E. coli*. This sophisticated experimental approach could not only open entirely new possibilities of recording, archiving, and propagating data but it could also be engineered further into an effective memory device. The properties of Cas1 and Cas2 that were engineered into the molecular recording tool, together with the novel understanding of the sequence requirements for optimal spacers, enables a significantly scaled-up potential for recording in the genome memories/molecular experiences cellular structures are having during their growth and development, or exposure to stresses and pathogens in a chronological fashion.

## Author Contributions

The author confirms being the sole contributor of this work and approved it for publication.

## Conflict of Interest Statement

The author declares that the research was conducted in the absence of any commercial or financial relationships that could be construed as a potential conflict of interest.
